# Curcumin-infused carboxymethyl cellulose–gelatin gel for oral tissue repair: Preliminary evaluation

**DOI:** 10.1016/j.jobcr.2025.08.023

**Published:** 2025-08-21

**Authors:** Senthil Rethinam

**Affiliations:** Nano-Bioproduct Research Lab (NBRL), Department of Pharmacology, Saveetha Dental College and Hospitals, Saveetha Institute of Medical and Technical Sciences (SIMATS), Saveetha University, Chennai, 600 077, Tamilnadu, India

**Keywords:** Oral drugs, Wound gel, Natural polymer, In vitro

## Abstract

**Background:**

This study aimed to develop and evaluate a multifunctional oral wound care gel (OWCG) formulated with carboxymethyl cellulose (CMC), gelatin (GEL), and curcumin (CUR) to enhance oral soft tissue regeneration.

**Methods:**

CMC was synthesized through etherification, whereas CUR was extracted using a modified ethanol-based method. OWCG was prepared and crosslinked using 5 % citric acid. Physicochemical characterization including Fourier-transform infrared (FTIR) spectroscopy, high-resolution scanning electron microscopy (HR-SEM), thermogravimetric analysis (TGA), swelling behavior, and in vitro drug release were assessed. Biological evaluations included in vitro anti-inflammatory, antibacterial, and MTT (3-[4,5-dimethylthiazol-2-yl]-2,5 diphenyl tetrazolium bromide) assays on fibroblast (3T3-L1) cell, and scratch wound healing assays.

**Results:**

OWCG exhibited a maximum swelling capacity of 86 % at neutral pH and 83 % CUR release under alkaline conditions within 24 h. In vitro protein denaturation inhibition was 82.4 %, confirming its anti-inflammatory efficacy. The gel exhibited notable antibacterial activity. MTT assays demonstrated over 90 % cell viability at 48 h, and scratch wound assays showed 90 % wound closure within the same timeframe.

**Conclusion:**

OWCG demonstrated good biocompatibility, antimicrobial efficacy, and wound healing potential, supporting its use in oral wound care.

## 1 Introduction

Oral wounds resulting from trauma, surgical procedures, infections, or underlying systemic diseases disrupt the delicate mucosal integrity and can significantly impair functions such as speech, mastication, and swallowing. Chronic oral lesions, such as those seen in patients with diabetes, oral cancers, or autoimmune conditions, pose unique challenges owing to constant moisture, microbial load, and mechanical irritation within the oral cavity. These wounds often experience delayed healing, leading to persistent discomfort, secondary infections, and reduced quality of life.^1^ In India, where access to specialized oral care is limited in many regions, the treatment of non-healing oral wounds can become both clinically and economically burdensome. Therefore, there is an urgent need to develop effective, affordable, and biocompatible oral wound care gels that can promote rapid healing, minimize infection, and improve patient outcomes.^2^

The wound healing process typically progresses through four sequential phases: hemostasis, inflammation, proliferation, and maturation. The hemostatic phase occurs immediately after injury, with the aim of preventing bleeding through vascular constriction and clot formation. During the inflammatory phase, immune cells clear pathogens and debris, establishing a clean wound bed.^3^ Various intrinsic and extrinsic factors—including moisture balance, infection, patient age, and overall body condition—can significantly influence the efficiency and outcome of wound healing.^4^

This study aimed to develop and evaluate an oral wound care gel (OWCG) with oxygen-releasing and antibacterial properties for potential applications in oral wound healing. The formulation was based on carboxymethyl cellulose (CMC), gelatin (GEL), and curcumin (CUR), which were selected for their biocompatibility, photo-crosslinking ability, and wound coverage efficiency. The primary objective was to enhance oral wound healing by promoting cell proliferation and reducing hypoxia-induced cell death through sustained oxygen release and antibacterial action. The secondary objective of this study was to assess the physicochemical properties, structural integrity, and potential anti-inflammatory effects of OWCG. The developed gel was designed to be portable, easily applicable to irregular or deep wounds, and capable of supporting cell migration, adhesion, and tissue regeneration.

## 2 Materials and methods

### 2.1 Preparation of carboxy methyl cellulose (CMC)

CMC was synthesized via etherification of purified cellulose. Initially, 10 g of cellulose was dispersed in 100 mL of isopropanol, followed by gradual addition of 20 mL of 30 % NaOH to activate the cellulose into alkali cellulose. After stirring for 1 h, 15 g of monochloroacetic acid (MCA) was added, and the reaction proceeded at 65 °C for 3 h. The mixture was then neutralized with glacial acetic acid, filtered, and thoroughly washed with 70 % and absolute ethanol to remove impurities and moisture. The resulting CMC was dried at 55 °C for 24 h, ground, and stored.

### 2.2 Preparation of gelatin (GEL)

GEL was extracted from cleaned and descaled fish skin using a hot water extraction method. Fish skin was first soaked in 0.1 M NaOH for 1 h to remove non-collagenous proteins, followed by thorough washing until a neutral pH was achieved. The pretreated skins was then subjected to acid swelling in 0.05 M acetic acid for 12 h. After rinsing, the swollen skins were heated in distilled water at 45–50 °C for 8 h to extract GEL. The GEL-rich solution was filtered, concentrated, and lyophilized to obtain dry GEL powder. For gel preparation, fish GEL was dissolved in warm distilled water (10 % w/v) at 40 °C with continuous stirring until a clear solution was formed, and then cooled to form a gel suitable for biomedical applications.

### 2.3 Preparation of curcumin

The extraction process was modified from that described in a previous study (Xiong et al., 2020). Ground turmeric (50 g) was extracted with 500 mL water and 70 % ethanol for 2 h at 100 °C. The extract was centrifuged at 6500×*g* at 4 °C for 10 min, and the supernatant was filtered through filter paper (No. 3, 110 mm, Whatman). The filtered solution was concentrated using a rotary evaporator under reduced pressure at 60 °C to remove ethanol.

### 2.4 Synthesis of oral wound care gel (OWCG)

The OWCG was prepared by dissolving 2.0 g of CMC, 0.5 g of GEL, and 1 mg of CUR in 100 mL of deionized water under continuous stirring at 200 rpm at room temperature until fully solubilized. Subsequently, 5.0 % (w/v) citric acid was added as a crosslinking agent, and the mixture was stirred at 400 rpm for 30 min to achieve uniform homogenization.

### 2.5 Statistical analysis

Statistical analyses were performed using the SPSS software, version 20.0 (SPSS, Inc., Chicago, IL, USA). One-way ANOVA was used to compare groups, and statistical significance was set at p ≤ 0.05.

## 3 Results

### 3.1 Physicochemical characterization

**Fig. 1:**
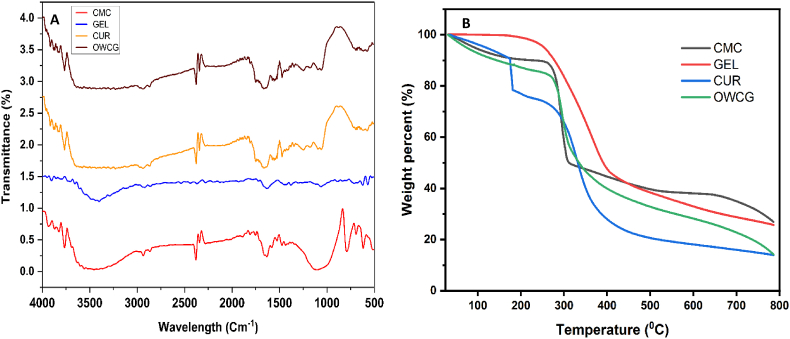
A: FTIR analysis of CMC, GEL, CUR, and OWCG B:TGA analysis of CMC, GEL, CUR, and OWCG, showing characteristic functional groups and confirming component integration.

In Fig. 1a show the functional groups identify the CMC, GEL, CUR, and OWCG, the FTIR spectrum of CMC displays characteristic peaks corresponding to hydroxyl, C–H stretching, and carboxylate groups, confirming successful carboxymethylation. Prominent bands at ∼1600 cm^−1^ and 1438 cm^−1^ are attributed to the asymmetric stretching of COO^−^ and symmetric stretching of COONa, respectively. The spectrum of GEL shows amide I, II, and III bands at ∼3100, 1640, 1550, and 1310 cm^−1^, indicating residual acetylated structures, which diminished due to deacetylation. The broad NH stretching bands (3270–3290 cm^−1^) and a distinct amide II peak at 1599 cm^−1^ confirm the presence of chitosan. CUR FTIR spectrum (4000–400 cm^−1^) reveals O–H stretching at 3506 cm^−1^, C=C/C=O bands at 1626 cm^−1^, and aromatic vibrations at 1495, 1454, and 1423 cm^−1^. Additional peaks at 1501, 1600, 1266, and 1149 cm^−1^ correspond to conjugated C=O and C–O stretching, validating curcumin's incorporation through its distinct vibrational signatures.

The thermal behavior of the composite films, as illustrated in Fig. 1b, was evaluated using TGA. CMC exhibited a residual weight of 34.2 % at 600 °C, indicating the presence of thermally stable, non-volatile components. The TGA curve of GEL displayed a typical three-step degradation pattern: initial moisture loss, followed by the breakdown of less stable amino acid chains, and finally the decomposition of more stable protein residues. Specifically, GEL showed three major weight-loss events at 81.57 °C, 160.70 °C, and 267.91 °C, corresponding to weight losses of 9.47 %, 6.82 %, and 70.34 %, respectively. In the case of CUR, significant thermal degradation was observed in the ranges 225–316 °C, 220–290 °C, and 190–230 °C, suggesting the progressive breakdown of its polyphenolic structure.

**Fig. 2:**
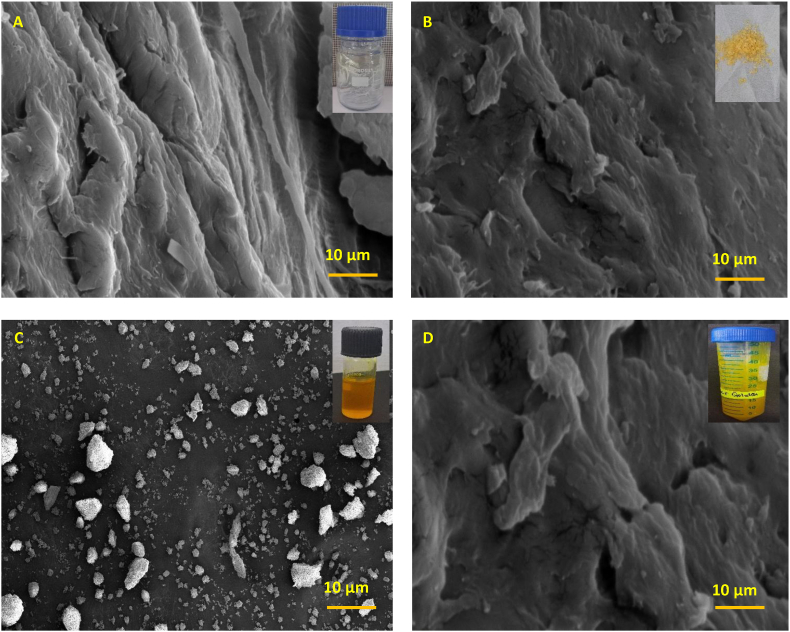
HRSEM images of A: CMC B: GEL C: CUR D: OWCG, highlighting surface morphology and structural integration of components.

HRSEM images of CMC, GEL, CUR, and OWCG formulations (Fig. 2a–d) revealed distinct differences in pore morphology and distribution. Pore size measurements, performed on randomly selected regions at varying magnifications, showed that both CMC and GEL exhibited irregular pore structures, with smaller pores concentrated at the periphery and larger ones toward the center, ranging from approximately 30 to 100 μm. The OWCG displayed a more heterogeneous surface, characterized by disorganized and variably sized pores with thick pore walls and occasional rough, and less porous areas. Notably, the OWCG incorporating CMC, GEL, and CUR exhibited a well-developed porous architecture with enhanced interconnectivity.

### 3.2 Swelling behaviour

**Fig. 3:**
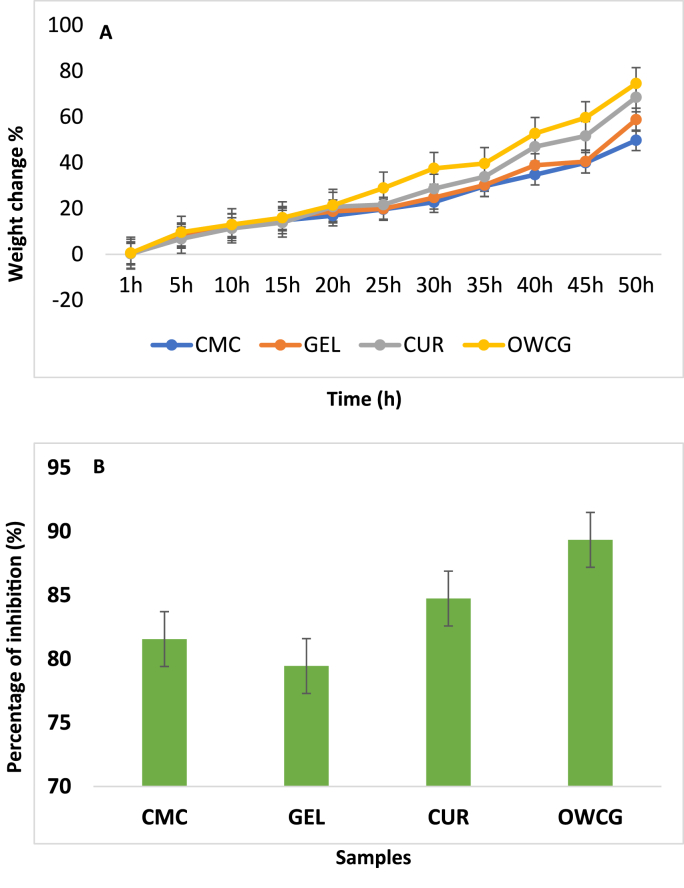
A**:** Swelling behaviour of CMC, GEL, CUR, and OWCG B: Anti-inflammatory Study of CMC, GEL, CUR, and OWCG.

As shown in Fig. 3a, CUR-loaded OWCG exhibited significantly higher swelling than the non-CUR formulations, particularly at 16 h (p = 0.017) and 20 h (p = 0.009), under neutral pH conditions. The swelling percentage reached ∼86 % in the CUR group versus ∼76 % in the control OWCG group (p < 0.05), indicating that CUR incorporation enhanced fluid uptake properties.

### 3.3 Anti-inflammatory study

CUR-loaded OWCG demonstrated a concentration-dependent inhibition of protein denaturation, with 82.4 % inhibition at the highest dose, which was statistically significant when compared to CMC and GEL alone (p = 0.021 and p = 0.037, respectively). The effect was comparable to that of diclofenac sodium (89.1 %, p = 0.091), suggesting strong anti-inflammatory potential (Fig. 3b).

### 3.4 Release profile

**Fig. 4:**
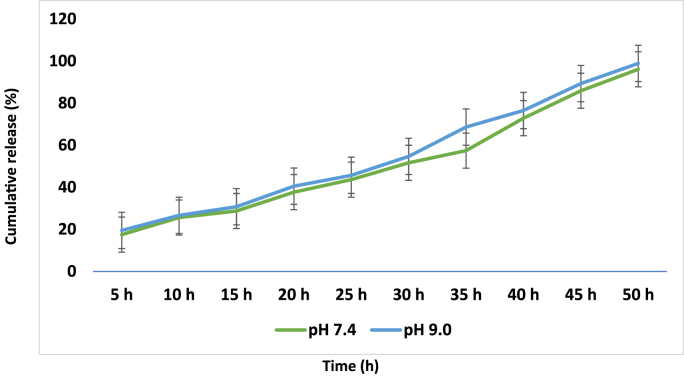
Drug release study of OWCG at different pH 7.4 and 9.0.

Cumulative drug release from CUR-loaded OWCG was significantly higher under alkaline conditions than under neutral conditions (p = 0.032), particularly within the first 16 h (Fig. 4). The enhanced release correlated with increased swelling and faster gel degradation.

### 3.5 Antibacterial activity

**Fig. 5:**
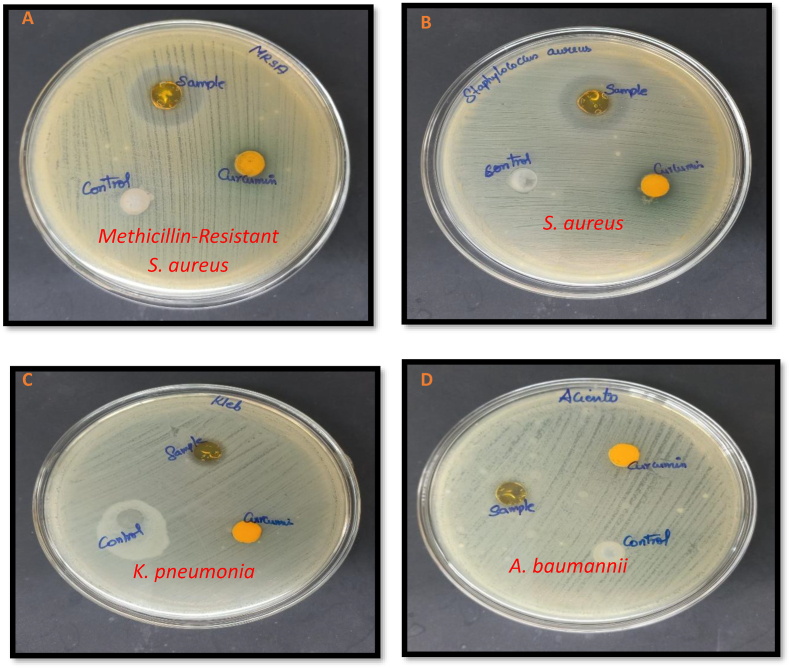
Antimicrobial activity- A: Inhibition zone of control (CMC), CUR, and OWCG against *Methicillin-Resistant S. aureus* B: Inhibition zone of control (CMC), CUR, and OWCG against *S. aureus* C: Inhibition zone of control (CMC), CUR, and OWCG against *k. pneumonia* D: Inhibition zone of control (CMC), CUR, and OWCG against *A. baumannii*.

As shown in Fig. 5(a–d), the OWCG demonstrated significantly larger zones of inhibition compared to individual components against *Methicillin-Resistant S. aureus* (38 ± 2.4 mm, p = 0.004) and *S. aureus* (36 ± 1.0 mm, p = 0.006). Similar statistically significant differences were observed against *K. pneumoniae* (14 ± 1.3 mm, p = 0.023) and *A. baumannii* (13 ± 2.2 mm, p = 0.031), confirming broad-spectrum antibacterial efficacy.

### 3.6 Cytotoxicity test

**Fig. 6:**
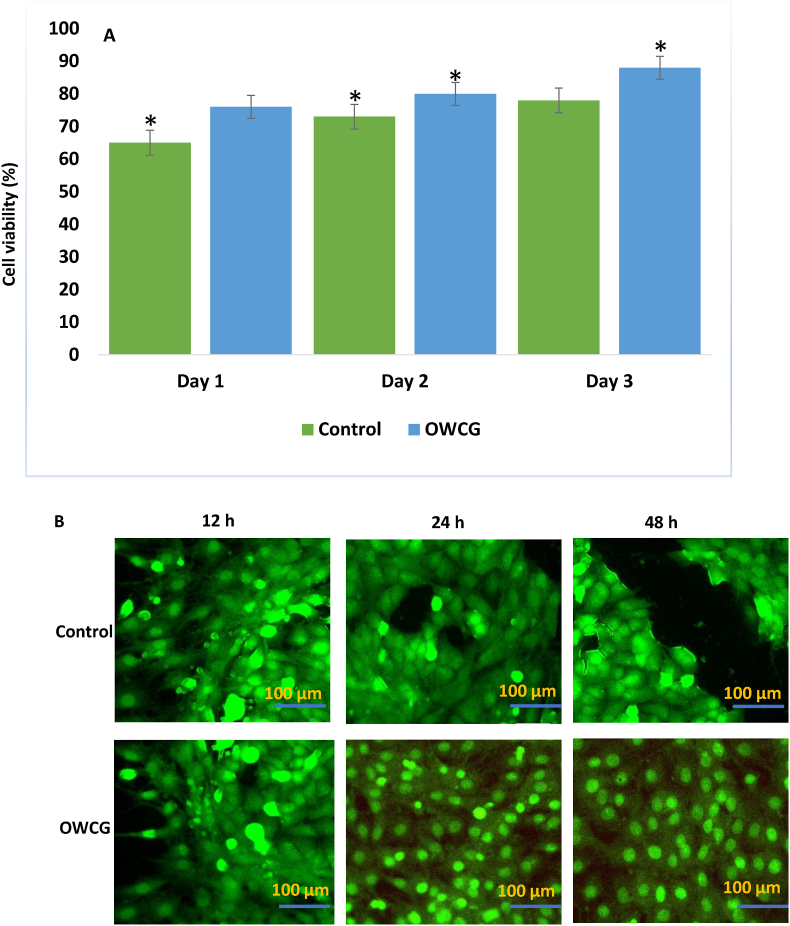
A: MTT assay demonstration control and OWCG on 3T3-L1 cells. The asterisks (∗) indicate statistically significant differences compared to the control p < 0.05 B: Fluorescence micrographs (20X) of HaCaT cells cultured (live cells) on 12, 24 and 48 h.

OWCG showed significantly higher cell viability at 24 and 48 h than CMC, GEL, and CUR (p < 0.05). After 48 h, cell viability in the OWCG group exceeded 85 %, compared to ∼65–70 % in the other groups (Fig. 6a), indicating superior biocompatibility. This was further supported by live staining (Fig. 6b), in which live cells fluoresced bright green.

### 3.7 In vivo investigation

**Fig. 7:**
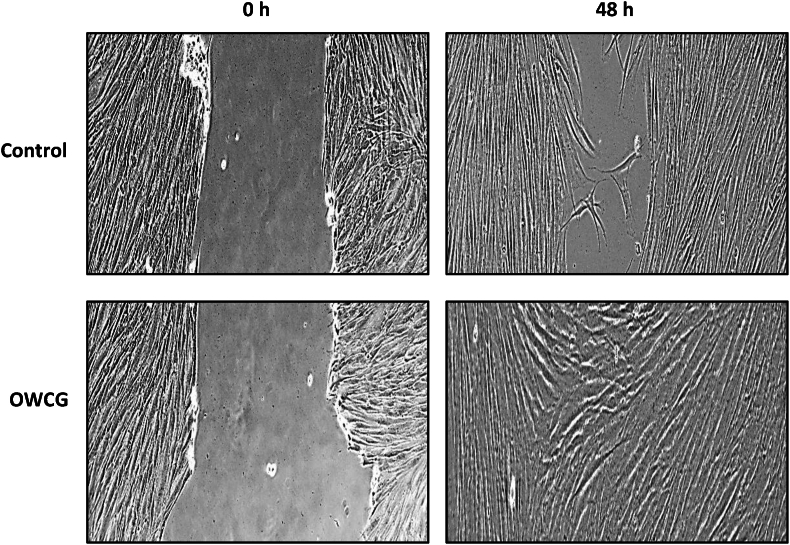
Cell migration assay of 3T3-L1 cells treated with control and OWCG.

The scratch wound assay revealed that OWCG significantly improved wound closure compared with the individual components. At 24 h, wound closure was ∼70 % in the OWCG group versus 50–60 % in the CMC and GEL groups (p = 0.013), and only ∼40 % in the CUR group (p = 0.007). At 48 h, the OWCG group reached approximately ∼90 % closure (p = 0.002), suggesting synergistic effects in promoting keratinocyte migration (Fig. 7).

## 4 Discussion

The present study developed and evaluated a novel oral wound care gel (OWCG) formulated using carboxymethyl cellulose (CMC), gelatin (GEL), and curcumin (CUR), with potential applications in oral and maxillofacial soft tissue repair. FTIR analysis confirmed successful incorporation and functional interactions among the composite components. These findings are consistent with those of previous reports on biopolymer-based therapeutic scaffolds for mucosal healing.^5^

Thermogravimetric analysis (TGA) revealed multi-phase degradation profiles for each component, with the OWCG exhibiting enhanced thermal stability attributable to the interaction among CMC, GEL, and CUR. This behavior supports the potential to maintain performance under physiological and handling conditions typical of oral surgical settings. Complementary HRSEM analysis demonstrated a uniformly porous microstructure in the OWCG, in contrast with the irregular pore networks of the individual components. This architecture facilitates oxygen exchange, exudate absorption, and controlled drug release all of which are critical for oral wound management, in which moisture retention and antimicrobial action are vital for epithelial regeneration.^6^

The swelling behavior of OWCG was strongly influenced by pH and polymer composition, showing maximum uptake under neutral and mildly alkaline conditions. These characteristics are advantageous in the oral cavity, where saliva pH fluctuations and dynamic fluid exchange require materials with responsive hydration and degradation profiles. CUR incorporation enhanced swelling and water retention, likely through disruption of internal hydrogen bonding and increased porosity.^7^

In vitro drug release studies indicated a biphasic release profile, with an initial burst followed by sustained diffusion. Notably, alkaline pH facilitated greater cumulative release, which could be therapeutically beneficial in infected or inflamed oral wounds characterized by elevated pH. Antibacterial studies further demonstrated that CUR-loaded OWCG exhibited superior inhibition of both Gram-positive and Gram-negative bacteria, suggesting a synergistic effect between the biopolymer matrix and CUR. This aligns with the current clinical needs for non-antibiotic wound care formulations that address biofilm-related complications.^8^

The MTT cytotoxicity assay on HaCaT keratinocytes confirmed the biocompatibility of OWCG, with enhanced cell viability observed after 48 h. The presence of GEL and CMC provided a conducive matrix for keratinocyte attachment and proliferation, whereas 10.13039/100020075CUR at the studied concentration supported antioxidant and anti-inflammatory responses without inducing cytotoxic effects. Moreover, protein denaturation assays indicated that OWCG significantly inhibited thermal denaturation of bovine serum albumin, a proxy for anti-inflammatory activity. Such effects are particularly relevant for minimizing inflammation-mediated tissue damage in post-extraction or post-biopsy.^9^

Recent studies have shown that nanofiber-based drug delivery systems, such as berberine chloride and curcumin-loaded nanofibers, effectively enhance wound healing and immune responses, particularly in resistant infections like MRSA.^10^ Meanwhile, nanoparticle-mediated therapeutic approaches are gaining momentum in cancer treatment owing to their targeted delivery and reduced toxicity. Additionally, the integration of machine learning in dental diagnostics is revolutionizing the early detection and classification of cysts, tumors, and abscesses, offering improved clinical decision-making.^11^ Biocomposites containing gelatin and polysaccharide derivatives have shown improved thermal and mechanical properties due to crosslinking.^12^ The porous structure observed in HRSEM supports prior findings on its role in moisture retention and drug diffusion.^13^ Curcumin's antimicrobial and anti-inflammatory effects in hydrogel-based wound systems further justify its inclusion in the OWCG formulation.^14^

Finally, OWCG promotes accelerated keratinocyte migration and wound closure, underscoring its potential utility in oral mucosal regeneration. The bioactive and structural properties of the gel mimic those of the extracellular matrix, offering a protective and stimulatory environment that is conducive to tissue repair. These findings support the translational potential of OWCG as a non-invasive, biodegradable, and efficacious biomaterial for oral wound management in maxillofacial surgery.

## 5 Limitation

Future studies should focus on long-term in vivo evaluations, clinical trials, and the incorporation of additional bioactive agents to further enhance therapeutic efficacy. However, the current limitations include the absence of large-scale clinical validation and potential variability in curcumin release due to pH sensitivity, which may affect consistency in diverse wound environments.

## 6 Conclusion

The CMC/GEL/CUR-based oral wound gel demonstrated excellent physicochemical stability, biocompatibility, and therapeutic performance. Its rapid swelling capacity, sustained drug release, significant antibacterial zones, and high cell viability confirmed its multifunctionality. The in vitro scratch wound assay on 3T3-L1 cells showed accelerated re-epithelialization, with 90 % wound closure at 48 h, highlighting its regenerative potential. Together, these findings suggest that the developed wound gel is a promising candidate for advanced wound care applications that, simultaneously support tissue regeneration, control inflammation, and prevent infections.

## Patient’s/guardian’s consent

Not applicable.

## Ethics approval and consent to participate

There are no animal/human subjects in this article.

## Source of funding

No funding was received for conducting this study.

## Declaration of competing interest

The authors declare that they have no known competing financial interests or personal relationships that could have appeared to influence the work reported in this paper.
